# Silicone Oil-Based Nanoadjuvants as Candidates for a New Formulation of Intranasal Vaccines

**DOI:** 10.3390/vaccines9030234

**Published:** 2021-03-08

**Authors:** Agnieszka Razim, Marcelina Pyclik, Katarzyna Pacyga, Sabina Górska, Jintao Xu, Michal A. Olszewski, Andrzej Gamian, Andrzej Myc

**Affiliations:** 1 Department of Microbiology, Hirszfeld Institute of Immunology and Experimental Therapy, Polish Academy of Sciences, 53-114 Wroclaw, Poland; marcelina.pyclik@hirszfeld.pl (M.P.); katarzyna.pacyga@hirszfeld.pl (K.P.); sabina.gorska@hirszfeld.pl (S.G.); 2 Division of Pulmonary and Critical Care Medicine, Department of Internal Medicine, University of Michigan Health System, Ann Arbor, MI 48109, USA; jintaoxu@med.umich.edu (J.X.); olszewsm@med.umich.edu (M.A.O.); 3 Research Service, Department of Veterans Affairs Ann Arbor Healthcare System, Ann Arbor, MI 48105, USA; 4 Department of Immunology of Infectious Diseases, Hirszfeld Institute of Immunology and Experimental Therapy, Polish Academy of Sciences, 53-114 Wroclaw, Poland; andrzej.gamian@hirszfeld.pl (A.G.); myca@umich.edu (A.M.); 5 MNIMBS, Department of Internal Medicine, University of Michigan, Ann Arbor, MI 48109, USA

**Keywords:** adjuvant, intranasal administration, cationic surfactant, emulsion, antigen, bioadhesion

## Abstract

Many conventional vaccines are administered via a needle injection, while most pathogens primarily invade the host via mucosal surfaces. Moreover, protective IgA antibodies are insufficiently induced by parenteral vaccines. Mucosal immunity induces both local and systemic response to pathogens and typically lasts for long periods of time. Therefore, vaccination via mucosal routes has been increasingly explored. However, mucosal vaccines require potent adjuvants to become efficacious. Despite many efforts to develop safe and robust adjuvants for mucosal vaccines, only a few have been approved for use in human formulations. The aim of our study was to design, develop and characterize new silicone oil-based nanoadjuvant candidates for intranasal vaccines with potential to become mucosal adjuvants. We have developed an array of nanoadjuvant candidates (NACs), based on well-defined ingredients. NAC1, 2 and 3 are based on silicone oil, but differ in the used detergents and organic solvents, which results in variations in their droplet size and zeta potential. NACs’ cytotoxicity, Tumor Necrosis Factor α (TNF-α) induction and their effect on antigen engulfment by immune cells were tested in vitro. Adjuvant properties of NACs were verified by intranasal vaccination of mice together with ovalbumin (OVA). NACs show remarkable stability and do not require any special storage conditions. They exhibit bio-adhesiveness and influence the degree of model protein engulfment by epithelial cells. Moreover, they induce high specific anti-OVA IgG antibody titers after two intranasal administrations. Nanoadjuvant candidates composed of silicone oil and cationic detergents are stable, exhibit remarkable adjuvant properties and can be used as adjuvants for intranasal immunization.

## 1. Introduction

Adjuvants are vaccine components that are designed to enhance the development of prolonged humoral and/or cell-mediated immune response to the target antigen. Effective adjuvant should possess at least one of the following features: to “depot” antigen, facilitate antigen presentation and antigen sampling by mucosal dendritic cells, activate/modulate the immune response, and induce the cytotoxic T lymphocytes [1]. During last 90 years, many new adjuvants have been developed but only a few of them reached clinical trials and only seven were approved by US. Food and Drug Administration for use in the human vaccine: Alum, AS01B, MF59™, virosome, AS03, ASO4 and CpG 1018 [2,3]. Mostly, they are used parenterally in influenza vaccines. ASO4 is used in hepatitis B virus (HBV) and human papilloma virus (HPV) vaccines [4]. CpG 1018 is used in HBV vaccine [5]. There are some prototypes of mucosal adjuvants under study, for example Endocine™ [6], CAF01 [7], nanoemulsion W805EC [8], GPI-0100 [9], CCS [10], cholera toxin B unit and *Escherichia coli* heat-labile toxin mutants or “hybrid” adjuvants containing cytokines [11,12,13,14,15]. Of particular interest are the nanoemulsion-based adjuvants, which have properties, such as droplet size and zeta potential that improve interactions with mucosa, and thereby, effectively induce an immune response [16]. Despite of remarkable research efforts, no universal adjuvant used for any kind of antigen and any route of administration has been obtained. Alum, that is widely used for parenteral immunizations, is unfit for mucosal vaccines since it does not induce mucosal immunity.

The development of comprehensive adjuvant(s) is a great challenge due to genetic and epigenetic heterogeneity of human beings. It must be safe in use on the high number of people to exclude variability of any side effects [17], and finally pass clinical trials. Moreover, adjuvanticity can be limited to some antigens [18]. Ultimately, in most cases, response to parenteral vaccination may be limited to systemic immunity, while mucosal immunity (or combination of both) may be more effective against the specific pathogens [19]. The intranasal vaccine offers a simpler and less invasive way of administration, higher patient compliance due to its straightforward application, and could be used for swift mass immunizations to combat sudden outbreaks and rapidly spreading infections [20]. However, an intranasal vaccine needs to overcome the natural barrier of the mucosal surface [21,22], which has high propensity to induce tolerance against applied antigens. Some of these obstacles could be eliminated by the application of vaccine that mimics charge and size of pathogens [22].

In the present study, we undertake the approach to use nanometer-size emulsion consisting of oil, nonionic and cationic surfactants, organic solvent and water called nanoemulsions developed by Myc, et al. [23]. The emulsion-making technology allowed us to further develop the new and different array of nanoemulsion formulations. From this, we characterized and selected three promising designs called nanoadjuvant candidates (NACs). The process of their production is relatively easy to perform and resize to large-scale manufacturing. NACs are long-term stable (at least a year) and they do not require to be frozen which is of the great contribution to the vaccine stability. On average, current vaccines must be used within two years when stored at 2–8 °C. It is crucial since any change in storage temperature may have a destructive effect on the vaccine efficacy. On practical side, this means that a significant part of the transported vaccines may not be effective [24]. Nanoadjuvants, in contrast to emulsion based on natural compounds like soybean oil, are based on silicone oil, in order to obtain better biological performance. Finally, potential ability of NACs to amplify Pathogen-Associated Molecular Pattern (PAMP) responses of the intranasal immunization in mouse model has been tested. To sum up, in this work we characterized the new adjuvant for intranasal vaccination, and we show preliminary in vivo data concerning systemic response induced after intranasal vaccination. The characteristics of mucosal response is ongoing.

## 2. Materials and Methods

### 2.1. Preparation of Nanoadjuvant Candidates

Three tested NACs were formulated using synthetic oil, cationic detergents, nonionic detergent, organic solvents, and ultrapure water (Table 1). Polydimethylsiloxane (PDMS, Sigma-Aldrich, Saint Louis, MI, USA, cat. no DMPS5X, mol wt ~3780 Da), used as NACs oil base (65% of NAC, *w*/*v*), is a silicon-based organic polymer, inert, non-toxic and widely used in cosmetic, pharmaceutical and food industry. PDMS is not mutagenic and is excreted unchanged in the feces [25]. Tyloxapol (Sigma-Aldrich) was used as nonionic surfactant (5% of NAC, *w*/*v*). It is well characterized and already used in food and cosmetic industry. For NACs formulation two cationic surfactants were used: cetylpiridinium bromide (CBP, Sigma-Aldrich) and benzyldimethyldodecyl ammonium chloride (BDMDDAC, Sigma-Aldrich) (1% of NAC, *w*/*v*). Other used ingredients were ethanol or acetone (8% of NAC, *w*/*v*) and ultrapure water (Gibco, Thermo Fisher Scientific, Waltham, MA, USA) used to dissolve cationic detergent, which is in the form of a powder (21% of NAC, *w*/*v*). Oil and the mixture of remaining components were preheated to 65 °C in two separate vials, mixed and emulsified by high sheer force mixer for 60 s at 10,000 rpm (Silverson, East Longmeadow, MA, USA) [23].

NACs are prepared as stated in the Table 1 are treated as 100% (neat concentration) concentrated and next were diluted with ultrapure water and stored in a refrigerator as 60% (*w*/*v*) stock solutions in rubber-sealed glass vials. Prepared NACs have white color and are opaque. NACs used in all of the following experiments were sourced from one production batch used within six months of preparation.

### 2.2. The Measurements of Size and Zeta Potential

NACs were characterized for the size of droplets by Dynamic Light Scattering (DLS) by measuring the z-average parameter. Zeta potential (ZP) was characterized by Electrophoretic Light Scattering (ELS) measurements. Both values were measured using Zetasizer Nano ZS (Malvern Panalytical, Malvern, UK) in Folded Capillary Zeta Cells (cat. DTS1070, Malvern Panalytical, Malvern, UK). Each measurement was performed five times (five measurements of the same dilution of a test sample) using automatic mode at 25 °C. Unless otherwise stated, NACs were diluted in 1 mM HEPES buffer pH 7 (Serva Electrophoresis GmbH, Heidelberg, Germany) to 0.1% just before the measurement.

### 2.3. Nanoadjuvant Candidates Long Term Stability

NACs were stored at 4 °C and observed up to 12 months for any sign of instability (disproportionation, creaming, sedimentation or coalescence). Size and ZPs were measured at time 0, 1, 2, 3, 4, 5, 6 and 12 months. The Shapiro Wilk test was applied to test changes in both size and ZP of NACs droplets stored for 12 months. The significance of the deviation from normal distribution (size and ZP) of NACs during the storage time was assigned based on the probability that the normal distribution was lower than 0.05 (*p* < 0.05) [26].

### 2.4. Mucoadhesion

Mucoadhesion was tested as previously described [27]. Mucin type III (porcine stomach, Sigma-Aldrich) was rehydrated for 30 min in 1 mM HEPES pH 7 at the concentration of 1 mg/mL. 0.1% NAC was incubated with or without mucin at the final protein concentration of 50 µg/mL for 5 min at RT prior to testing. Interaction of NACs with mucin was assessed by measurement of droplets size and ZP with and without mucin. Each measurement was repeated five times (five measurements of the same dilution of a test sample). Experiment was repeated two times.

### 2.5. Isolation of BMDMs and Cell Cultures

All cell culture media and additives were purchased from Gibco, Thermo Fisher Scientific. Bone-marrow-derived macrophages (BMDM) were generated from C57BL/6 SPF mice and cultured, as previously described [28]. Briefly, bone marrow cells were harvested from flushed marrow cavities of femurs and tibiae of mice under aseptic conditions and were cultured (37 °C, 5% CO_2_) in complete medium consisting of RPMI supplemented with 10% FBS and 50 ng/mL M-CSF (Peprotech, Rocky Hill, CT, USA). After 7 days of incubation, the nonadherent cells were discarded, and adherent cells were removed by cell scraping following incubation on ice for 20 min in PBS. Cells were counted, aliquoted and frozen in liquid nitrogen in complete medium with 10% DMSO to store for later use.

TC-1 cell line (ATCC^®^ CRL-2785™, *Mus musculus* lung epithelial cell line) was obtained from HIIET PAS collection (Wroclaw, Poland). Cells were cultured in complete medium consisting of DMEM medium supplemented with 10% FBS. TC-1 cell line was used in our study as a model of airway epithelium, as it was previously used in other studies [16,29].

JAWS II cell line (ATCC^®^ CRL-11904™, *Mus musculus* immature dendritic cells) was obtained from HIIET PAS collection (Wroclaw, Poland). Cells were cultured in complete medium consisting of α-MEM supplemented with ribonucleosides and deoxyribonucleosides, 4 mM L-glutamine, 1 mM sodium pyruvate and 5 ng/mL murine GM-CSF and 10% FBS.

### 2.6. The Testing of Nanoadjuvant Candidates’ Cytotoxicity

BMDMs, TC-1 or JAWS II cells (2 × 10^4^ cells/well) were seeded on a 96-well plate in 100 µL of complete medium and incubated overnight (37 °C and 5% CO_2_). One hundred µL of 2× concentrated NAC was added to obtain the desired final concentration (from 0.001% to 0.1%). Cells were incubated for 8 and 24 h. The cytotoxicity was measured using SRB (Sulforhodamine B, Sigma-Aldrich) colorimetric assay, as previously published [30]. The plates were read at 510 nm wavelength by a SpectraMax plate reader (Molecular Devices, San Jose, CA, USA). Untreated cells were used as negative control (as 100% of live cells). Each treatment was done in triplicate.

### 2.7. Induction of TNF-α

BMDMs (0.1 × 10^5^ cells/well) were seeded on 96-well plate in 200 µL of 10% FBS complete medium and incubated overnight. Next day the medium was replaced with 180 µL of fresh medium. Ten µL of tested NAC was added to obtain the final concentration of 0.001% and 0.003%. Ten µL of 20 × concentrated lipopolysaccharide (LPS, *E. coli* K12, InvivoGen, San Diego, CA, USA) was added to each well containing NAC and to reference control well (without NAC) to obtain the final concentration of 1 ng/mL, which is suboptimal for cell activation. As a negative control, BMDMs were incubated with NAC alone. Cells were incubated for 24 h and supernatants were collected for cytokine quantification. A 24 h time point was selected as a maximum contact time of nanoemulsion and nasal mucosa. It is based on the finding that after 18 h post intranasal vaccination there were only traceable amounts of nanoemulsion + QDOT in mouse nostrils [16]. TNF-α was measured using ELISA MAX Mouse TNF-α set (BioLegend, San Diego, CA, USA). Other cytokines tested by us were IL-10 and IL-6 (DuoSet ELISA, R&D Systems, Minneapolis, MN, USA). Measurements were done following manufacturer protocol; each measurement was done in duplicate. The experiment was repeated 4 times.

### 2.8. Expression of TLR4

BMDM cells (1 × 10^6^ TC-1) were seeded on a 6-well plate in 750 µL of 10% FBS complete medium and incubated for 4 h. Next, 250 µL of NACs+/− ultrapure LPS in sub-activating concentrations (1 ng) were added in complete medium. Cells were incubated overnight and subsequently used for mRNA analysis in terms of TLR4 expression. Untreated cells were used as negative control.

### 2.9. RT-qPCR Analysis

The total RNA was isolated by TRI Reagent^®^ (Sigma Aldrich) and chloroform followed by isopropanol precipitation. The obtained pellet was washed with 75% EtOH (Ethanol pure, 99.8%; Chempur) and dissolved in UltraPure™ water (Invitrogen). RNA concentration was measured at 260 nm and 260/280 ratio in a BioPhotometer (Eppendorf AG, Germany). Reverse transcription was performed using M-MLV Reverse Transcriptase (M-MLV Reverse Transcriptase kit; Promega). cDNA was stored at −20 °C until further used. To check gene expression of TLR4 receptors, two pairs of starters were used: TLR4 RM/TLR4 FM (5′ ACAGCCACCAGATTCTCTAAAC 3′/5′ GCTTACACCACCTCTCAAACT 3′) (Sigma Aldrich) for TLR4 receptor gene and RM1_Actb/FM1_Actb (5′ TGTGCACTTTTATTGGTCTC 3′/5′ GATGTATGAAGGCTTTGGTC 3′) (KiCqStart^®^SYBR^®^ Green Primers; Sigma Aldrich) for a reference gene (β-actin). The standard PCR program was used in Bio-Rad CFX ConnectTM Real Time System (BioRad, Hercules, Clearwater, FL, USA). After each Real-Time PCR experiment the melt curve analysis was performed (0.5 °C for 5 s, 65–95 °C). Results are presented as the mean ± SD (*n* = 4). Before plotting these data on a graph, it was normalized to β-actin expression and related to a negative control (negative control corresponds to 0).

### 2.10. DQ-OVA Engulfment

The measurement of DQ-OVA engulfment by TC-1, JAWS II and BMDM cells was performed as previously published [29]. Briefly, cells (0.2 × 10^6^ cells/well) were seeded on 24-well plate in 500 µL of complete medium with 10% FBS and incubated for 4 h with or without 0.03% or 0.06% NAC and 10 µg/mL DQ-OVA which is a fluorogenic substrate for proteases and works according to vendor’s instruction (cat. D12053, Thermo Fisher Scientific). NACs concentrations were chosen based on the cytotoxicity assay results in such a manner that the sub-cytotoxic range of concentration were used. Before trypsinization, cells were washed twice with warm PBS to remove residues of NAC, OVA, cell debris and detached dead cells. Cells were then analyzed with FACSCalibur Cell Analyzer (BD Biosciences, Franklin Lakes, NJ, USA). Each sample was prepared in triplicate. Results shown are representative of at least two independent experiments.

### 2.11. The Interaction between OVA and Nanoadjuvant Candidates

The interaction between protein and NACs was studied by size and ZP measurements of their mixture. Briefly, OVA (grade V, Sigma-Aldrich) was rehydrated at 10 mg/mL in 1 mM HEPES pH 7 at RT for 30 min and added to 60% NAC, to a final concentration of 1 mg/mL OVA and 20% NACs. Before measurements, the mixture was diluted to the 0.1% NAC concentration. As a negative control NACs alone were used. Each measurement was repeated five times (five measurements of the same dilution of a test sample). Experiment was repeated two times. Protein concentrations were measured using Pierce BCA Protein Assay Kit from Thermo Fisher Scientific.

### 2.12. Mice Immunization

Pathogen-free, 6-week-old female C57BL/6 mice (Janvier Labs, Le Genest-Saint-Isle, France) were housed under standard laboratory conditions (22 °C, 12 h light/dark cycle, with ad libitum access to food and water). All procedures were approved by the Local Committee on the Use and Care of Animals in Wroclaw (approval no. 067/2019) and were performed in accordance with these guidelines. C57BL/6 mice (*n* = 5 per group) were vaccinated intranasally (IN) twice, four weeks apart with 12 µL (6 µL/nare) of a formulation consisting of 20 µg OVA/mouse with 20% NAC or without (PBS only) as previously described [31]. For vaccination, mice were sedated with 2.5% isoflurane and vaccinated in supine position. OVA used for immunizations was obtained from Sigma-Aldrich (99% pure), hydrated in PBS pH 7.4 and sterile-filtered through 0.22 µm filter before mixed with NAC.

Blood samples were taken after two weeks of each immunization from the facial vein. Samples were then allowed to clot at 37 °C and after approximately 1 h were spun to obtain the serum. Sera of each group were pooled. OVA-specific serum IgG, IgG types (IgG1, IgG2c), IgA and IgE were measured using specific secondary antibodies conjugated with HRP (Bethyl Laboratories, Montgomery, AL, USA IgG cat. E90-131, IgG1 cat. E90-105, IgA cat. E90-103, IgE cat. E90-115; ThermoFisher Scientific IgG2c cat. PA129288) and 96-well plates (MaxiSorp, Thermo Fisher Scientific) coated with 10 µg/mL OVA in carbonate/bicarbonate buffer pH 9.6 as in the manufacturer protocol. Serum anti-OVA IgE were measured in pooled serum samples diluted 1:10. Optical density (OD) was measured at 450 nm with the correction at 570 nm. Endpoint titer is the dilution of serum which when tested in ELISA gave ODs three times higher than the one obtained for PBS-treated controls [23].

### 2.13. Statistical Analysis

Microsoft Excel 2016 and GraphPad Prism 8 were used for graphing and statistical analyses. Comparisons were made between groups using *t*-test, one-way or two-way ANOVA. Shapiro-Wilk test was used as a normality test (*p* < 0.05).

## 3. Results

### 3.1. Physicochemical Parameters and Stability of Nanoadjuvant Candidates

Preliminary studies of formulations based on silicone oil and a mixture of cationic and non-ionic detergents have allowed the formation of a series of nanoadjuvant candidates (NACs). Owing to their remarkable stability, we have selected three of them (designated as NAC1, NAC2 and NAC3) and subjected to detailed characterization. We specifically focused on droplet size and ZP as these parameters are crucial for biological interactions like bioadhesion and particle engulfment by immune cells [32,33,34]. Table 1 lists all components used in each NAC. Physicochemical characteristics of NACs is listed in Table 2 (at timepoint 0). The polydispersity index (PDI) represents the droplets heterogeneity [35]. NAC1 and 2 are moderately dispersed and NAC3 is highly dispersed. This notable correlation between droplet size and polydispersity of NACs was consistent with basic principle of fluid mechanics that larger emulsion droplets would also display greater variation size [36]. Likewise, the ranges of charges reflected the properties of the cationic detergents used for NAC generation, resulting in ZP ranging from approximately 30 to 70 mV (Table 2, at timepoint 0).

We evaluated NACs stability in size (Table 2, (A)) and ZP (Table 2, (B)) during 12 months of storage, NACs were measured at each time point (0, 1, 2, 3, 4, 5, 6 and 12 month of storage) and the data was analyzed with Shapiro-Wilk normality test (*p* < 0.05). All the NACs passed the normality test indicating that they are stable in size and ZP during 12 months of storage. Therefore, all NAC1-3 displayed optimal and stable physicochemical properties consistent with a durable, emulsion-based adjuvant suitable for mucosal applications and therefore we preferred them for further investigation.

### 3.2. Nanoadjuvant Candidates Adhere to Mucin

**Figure 1:**
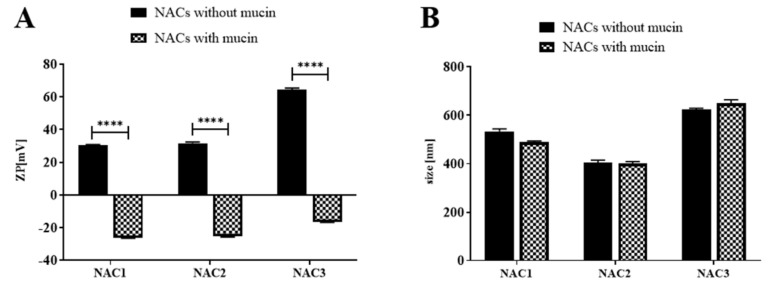
NACs interaction with mucin. (**A**) shows changes in ZP after mucin addition. (**B**) shows changes in size after mucin addition. Mucin type III was rehydrated for 30 min in 1 mM HEPES pH 7 at the concentration of 1 mg/mL. 0.1% NAC was incubated with or without mucin at the final protein concentration of 50 µg/mL for 5 min at RT prior to testing. Means from five measurements of each sample with ±SD, experiment was repeated twice, data pooled and analyzed with two-way ANOVA (multiple comparisons, corrected with Šidák test recommended by GraphPad Prism 8); *p* < 0.0001 (****).

It was already shown that interaction with mucin enhances the effectivity of potential mucosal vaccines [37]. Mucins are highly glycosylated, hydrophilic, negatively charged proteins that form a physical barrier on the surface of mucous membranes, which protect epithelial cells from the potential damage induced by external factors. We tested the interaction of NACs 1-3 with mucin in terms of changing ZP and nanodroplet size as described above. Size and ZP of the droplets were measured after mucin addition. All three NACs responded with the change of their ZP from positive to negative values (Figure 1A) with no changes in their size (Figure 1B). Droplets of NAC3, which have the highest ZP, exhibited the biggest change in ZP upon mucin addition (ΔZP = 80.78 mV). ZPs of NAC1 and 2 changed in similar extent (ΔZP = 56.46 mV and 56.96 mV, respectively). Thus, considering the change of ZP only, we conclude that all tested NACs are interacting with mucin via electrostatic binding.

### 3.3. The Effect of NACs on Viability of the Cells

To allow for future use NACs in vaccines, we determined cell tolerance to the increasing NAC1-3 concentrations and established their cytotoxic limits. These established concentrations were used in further in vitro experiments. Three cell types were selected for these studies: Airway epithelia, which form the mucosal barriers throughout the body (TC-1 cell line), dendritic cells (JAWS II) and macrophages (BMDMs), representing phagocyte/antigen-presenting cell group “patrolling” mucosal surfaces (e.g., lungs) and contributing to mucosal immunity. The three cell types were treated with NACs 1-3 at the concentrations of 0.001%, 0.003%, 0.01%, 0.03% and 0.1% for 8 and 24 h. NAC concentrations tolerated by more than 70% of cells following the incubation were defined as sub-cytotoxic. It was previously shown that such sub-cytotoxic effect leads to the induction of danger signals important for vaccine efficacy [38]. The NAC untreated cells served as a negative control (100% of live cells).

**Figure 2:**
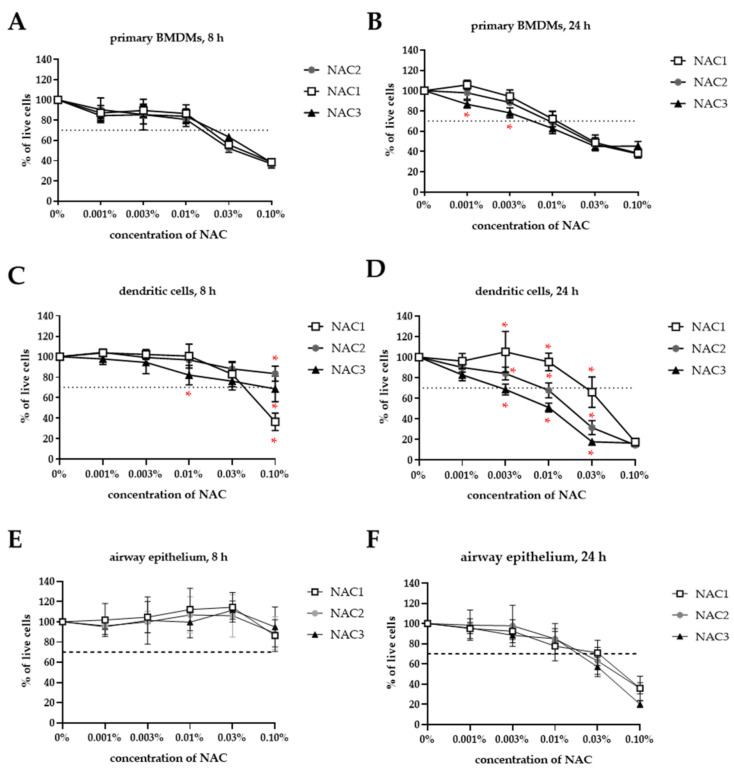
Determination of NAC concentration cytotoxicity. (**A**) Shows NACs effect on BMDMs viability after 8 h and (**B**) 24 h incubation. (**C**) shows NACs effect on dendritic cells viability after 8 h; and (**D**) 24 h incubation; (**E**) shows NACs effect on TC-1 cells viability after 8 h and (**F**) 24 h incubation. Cells (2 × 10^4^ cells/well) were seeded on a 96-well plate in 100 µL of complete medium and incubated overnight (37 °C and 5% CO_2_). One hundred µL of 2× concentrated NAC was added to obtain the desired final concentration (from 0.001% to 0.1%). The cytotoxicity was measured using SRB (Sulforhodamine B) colorimetric assay, Dotted line indicates 70% cytotoxicity. Data is shown as means with ±SD and was analyzed with two-way ANOVA (multiple comparisons, corrected with Tukey test recommended by GraphPad Prism 8); *p* < 0.0332 (*). Red asterisks indicate statistically significant differences calculated on three independent experiments.

All three NACs tested demonstrated similar tolerable versus cytotoxic concentrations (Figure 2A–F) except for dendritic cells treated for 24 h. The highest NAC concentration that was well tolerated by 70% of BMDMs was 0.01% for 8 h incubation and 0.003% for 24 h incubation. In case of BMDMs NAC3 exhibited significantly higher cytotoxicity at lower concentrations (0.001% vs. 0.003%) compared to NAC1 and NAC2 during 24 h incubation. Dendritic cells show an intermediate level of resistance against NAC during 8 h of incubation, 0.03% of NAC is nontoxic. 24 h incubation time is the most differentiating for NACs in case of dendritic cells. NAC3 shows a strong cytotoxic effect even at low concentrations (0.003%). The epithelial cells showed the greatest tolerance to high NAC concentrations. All three NACs of up to 0.01% were well-tolerated by over 70% of epithelial cells during 24 h incubation period. Of particular interest, none of the tested concentrations showed cytotoxic effect on epithelial cell lines when incubated for 8 h (Figure 2E), suggesting that epithelial cells are more resistant to NACs than macrophages and dendritic cells, at least within the tested range of NAC concentrations.

### 3.4. Nanoadjuvant Candidates Potentiate TNF-α Response to LPS Treatment

**Figure 3:**
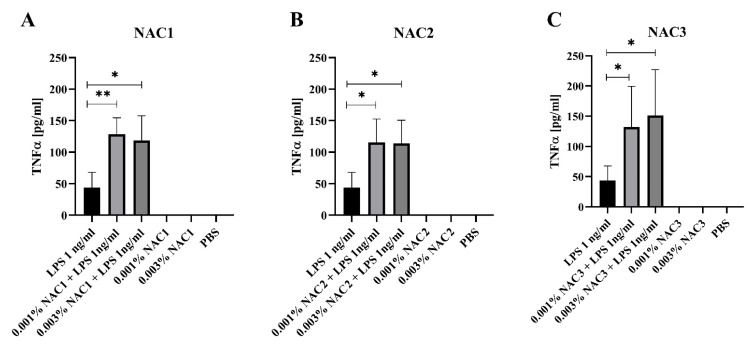
Co-stimulation effect of NACs (0.001% or 0.003%) and sub-activating concentrations of LPS on BMDMs. (**A**) shows the effect of NAC1; (**B**) shows the effect of NAC2; (**C**) shows the effect of NAC3. BMDMs (0.1 × 10^5^ cells/well) were seeded on 96-well plate and incubated with NACs final concentration of 0.001% and 0.003% with or without 1 ng/mL LPS. As a negative control BMDMs were incubated with PBS. Cells were incubated for 24 h and supernatants were collected for cytokine quantification. Cytokine levels were estimated in the supernatants using TNF-α ELISA kit (BioLegend). Experiment was repeated four times. Data was shown as means with ±SD and were analyzed with *t*-test (GraphPad Prism 8); *p* < 0.0332 (*), *p* < 0.0021 (**).

One of the functions of adjuvant along with antigen “delivery” is promoting danger signal responses, which in turn facilitate the development of an appropriate immune response as opposed to the immune tolerance [39]. We studied whether NACs promote danger signal response, using a TNF-α cytokine production as a readout of a danger signal-response of BMDMs. We applied a threshold concentration of LPS (1 ng/mL) as a model PAMP trigger of a borderline TNF-α response. While none of the tested NACs alone induced this cytokine production by BMDMs, the combination of each NAC with the threshold dose of LPS led to significantly greater induction of TNF-α by BMDM cells (Figure 3A–C). Interestingly, all 3 NACs worked with similar potency increasing TNF-α production by nearly 3-fold at 0.001% NAC concentration. Thus, all 3 NACs acted as effective enhancers of the danger signal response. We did not detect significant changes in IL-6 and IL-10 cytokine levels between tested groups.

### 3.5. Nanoadjuvant Candidates Induce Expression of TLR4 in Airway Epithelial Cells

**Figure 4:**
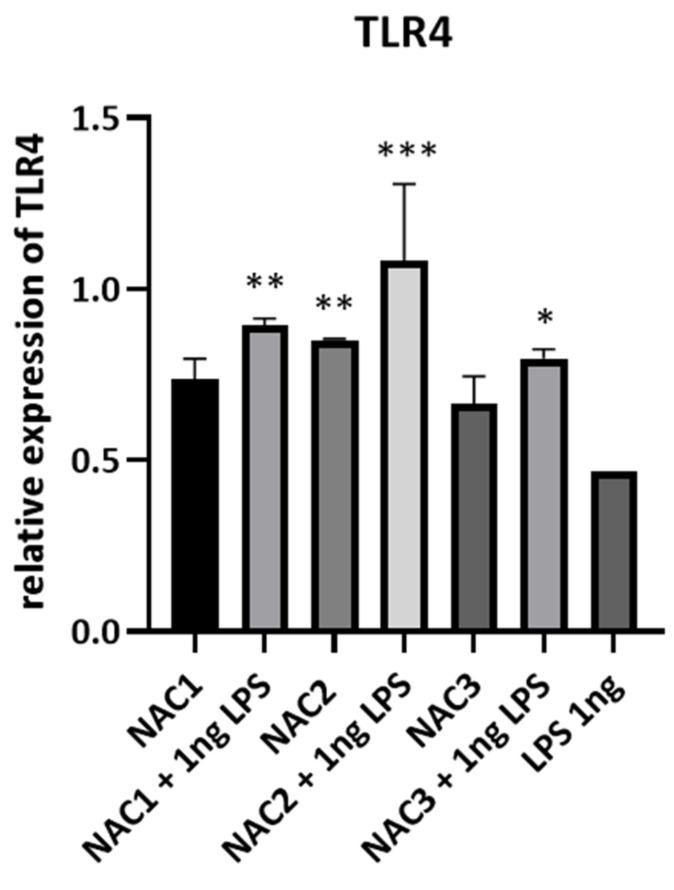
Induction of expression of TLR4 by NACs+/− LPS. 1 × 10^6^ TC-1 cells were stimulated with 0.003% NACs with or without 1 ng of LPS overnight. As negative control were used untreated TC-1 cells. Cells treated with 1 ng LPS were used as positive control. Total RNA was extracted and analyzed with RT-qPCR as described in M&M section. Experiment was repeated 4 times, data shown as means ±SD normalized to β-actin and relative to negative control (relative expression of TLR4 in negative control equals 0). Data is analyzed with two-way ANOVA (multiple comparisons, corrected with Dunnett test recommended by GraphPad Prism 8) comparing the treatment with NAC, NAC + LPS to LPS only; *p* < 0.0332 (*), *p* < 0.0021 (**), *p* < 0.0002 (***).

TLR4 agonists were already shown to be potent immune stimulators useful in vaccine preparations [40]. In order to asses NACs influence on TLR4 expression we stimulated airway epithelial cells with NACs+/− LPS. The RT-qPCR analysis has shown that NACs have an enhancing effect when added together with sub-activating concentrations of LPS when compared to LPS alone (Figure 4). There is a visible induction of TLR4 expression when NACs are added alone to the cells but this effect was not statistically significant. No similar effect was noted in case of macrophages (data not shown).

### 3.6. Nanoadjuvant Candidates Facilitate Protein Sampling by Airway Epithelial and Dendritic Cells

**Figure 5:**
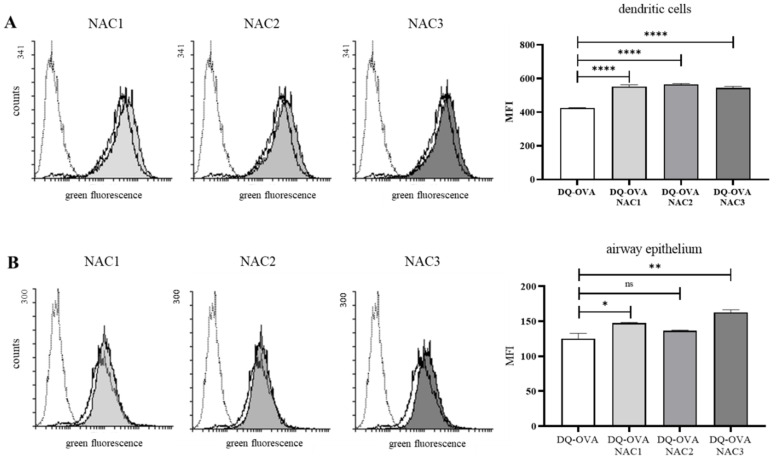
The effect of NACs on antigen engulfment by dendritic (**A**) and nasal epithelial cells (**B**). Cells (0.2 × 10^6^ cells/well) were seeded on 24-well plate in 500 µL of complete medium with 10% FBS and incubated for 4 h with or without 0.03% or 0.06% NAC and 10 µg/mL DQ-OVA. After incubation, cells were washed twice with warm PBS and detached by trypsinization. Cells were then analyzed with FACSCalibur Cell Analyzer (BD Biosciences). (**A**) shows cytofluorimetric histograms of the engulfment of DQ-OVA by dendritic cells. Dotted-line white histograms are non-treated controls, white histograms are dendritic cells incubated with DQ-OVA (10 µg/mL), grey histograms are dendritic cells incubated with DQ-OVA and 0.03% NAC. MFIs are presented as a bar graph means with ±SD, data analyzed one way ANOVA (*p* < 0.05) (multiple comparisons, corrected with Dunnett test recommended by GraphPad Prism 8). (**B**) shows cytofluorimetric histograms of the engulfment of DQ-OVA by TC-1 cells. Dotted-line white histograms are non-treated controls, white histograms are TC-1 cells incubated with DQ-OVA (10 µg/mL), grey histograms are TC-1 cells incubated with DQ-OVA and 0.06% NAC. MFIs are presented as a bar graph means with ±SD, data analyzed with one way ANOVA (multiple comparisons, corrected with Dunnett test recommended by GraphPad Prism 8); *p* < 0.0332 (*), *p* < 0.0021 (**), *p* < 0.0001 (****); ns means no significance.

The tight epithelial cell lining at the mucosal surfaces of the nose creates a natural barrier limiting antigen transfer into immune cells, of which majority remains submerged below the epithelial layer with some dendritic cells able to penetrate the layer and sample the antigen. Therefore, antigen transport through the epithelial barriers is an important step in generation of robust immune responses [41]. We then asked whether addition of NACs would facilitate protein uptake by macrophages, dendritic and epithelial cells. Protein uptake by mouse dendritic cells was measured using flow cytometry. DQ-OVA used as antigenic protein in our model, becomes fluorescent upon processing by intracellular proteases, providing evidence of its engulfment by the immune cells. All of the tested NACs in the concentration of 0.03% significantly enhanced the antigen sampling by dendritic cells (Figure 5A). We performed similar analysis with epithelial cells but since epithelial cells are more resistant to NACs that was shown on Figure 2 we used higher NACs concentration that was 0.06%. While 4 h incubation of DQ-OVA alone with the epithelial cells resulted in a robust uptake of the protein (increased Mean Fluorescence Intensity (MFI) to 125.31 FU) in the presence of NAC1, NAC2 and NAC3, we found enhanced MFI values of 147.37, 136.46, and 162.87 FU, respectively (Figure 5B). These differences were statistically significant in the case of NAC1 and NAC3. NAC3 (size 623.6 nm, ZP 64.4 mV) also induced the antigen uptake to a greater extent than NAC1 (size 534 nm, ZP 30.4 mV). Therefore, NAC1 and NAC3 substantially facilitated active transport of protein antigen into airway epithelial cells. It was not possible to demonstrate an effect on antigen uptake in macrophages as the antigen was actually immediately absorbed by these cells under the experimental conditions.

### 3.7. Interaction of Nanoadjuvant Candidates with OVA

**Figure 6:**
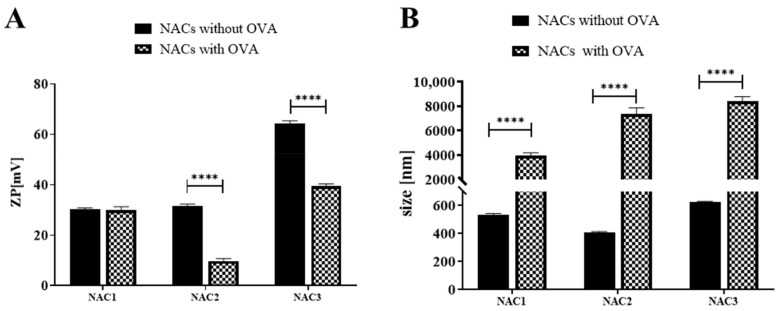
NACs interaction with OVA. Shows changes in ZP (**A**) and in size (**B**) after OVA addition. OVA was rehydrated at 10 mg/mL in 1 mM HEPES pH 7 at RT for 30 min and added to 60% NAC, to a final concentration of 1 mg/mL OVA and 20% NACs. Before measurements, the mixture was diluted to the 0.1% NAC concentration. As a negative control NACs alone were used. Means from five measurements of each sample with ±SD, experiment was repeated twice, data pooled and analyzed with two-way ANOVA (multiple comparisons, corrected with Šidák test recommended by GraphPad Prism 8); *p* < 0.0001 (****).

One of the properties of adjuvants is their interaction with antigens. OVA is a widely used model antigen in immunology/immunization studies. At pH 7 OVA is charged negatively (pH > pI, pI = 4.5) [42]. Thus, we evaluated interaction of NACs with OVA by measuring how “pulsing” each NAC1-3 with OVA affects their physicochemical parameters. Changes in droplet size and ZP were evaluated as described in M&M. In case of NAC1 there was no change in ZP after OVA addition, while NAC2 and NAC3 showed a substantial ZP reduction, albeit it remained positive (Figure 6A). All tested NACs enlarged their droplet size after mixing with OVA indicating presence of physical interaction between NAC1-3 and OVA (Figure 6B). The size of the NAC1-OVA droplets was the smallest while those of NAC3-OVA the biggest. However, the obtained PDIs were high (close to 1) which indicates very high particle heterogeneity which may influence data quality.

### 3.8. Nanoadjuvant Candidates Induce High Titer of Anti-OVA IgG Antibodies

**Figure 7:**
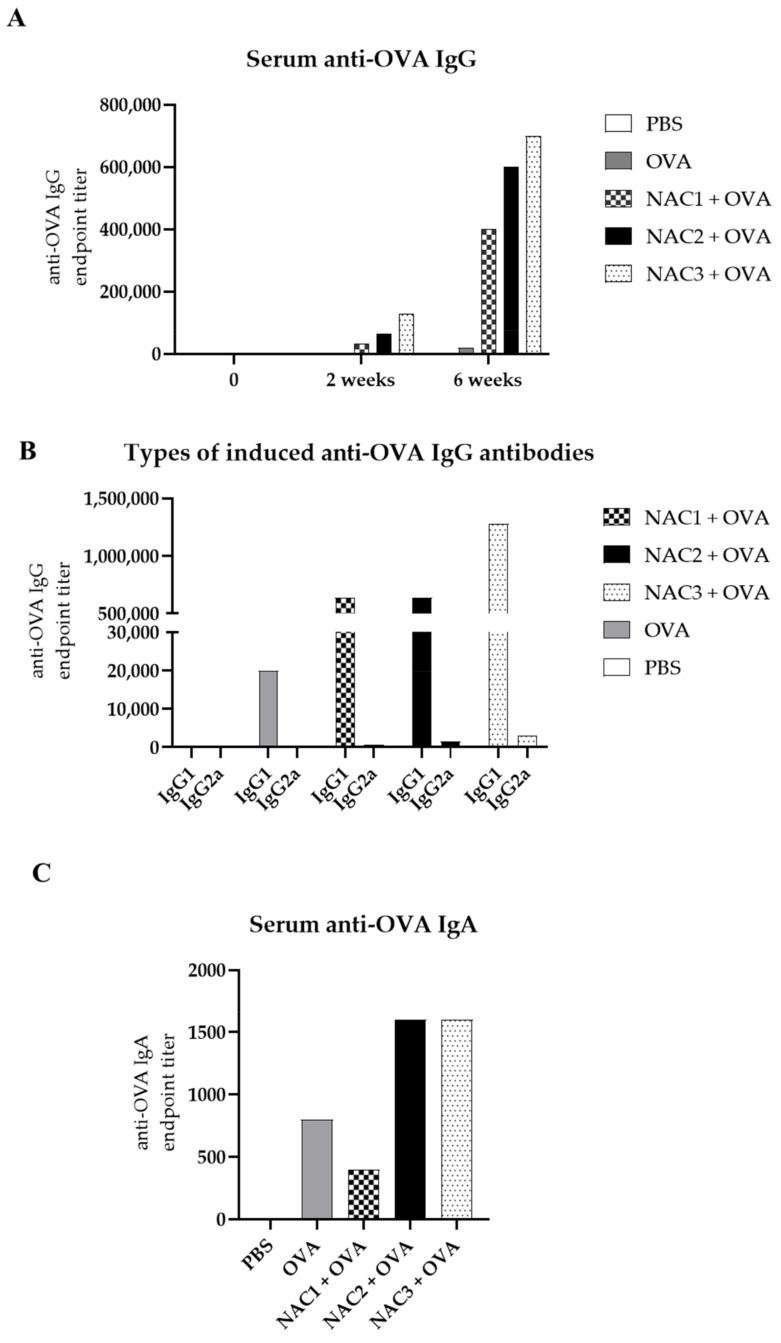
Serum anti-OVA antibody titer. Mice were immunized at time 0 and four weeks later. Sera of mice (*n* = 5) in each group were pooled. (**A**) shows endpoint anti-OVA IgG titer for pooled sera in each group at time 0, two weeks after first immunization and two weeks after second immunization. (**B**) shows titer of IgG1 and IgG2c types of antibodies measured in pooled sera two weeks after second immunization. (**C**) shows endpoint anti-OVA IgA titer measured in pooled sera two weeks after second immunization. Endpoint titers were estimated using ELISA kits (Bethyl Lab). Endpoint titer is defined as the reciprocal of the highest analyte dilution that gives a reading above the cutoff value (three times higher ODs than the one obtained for PBS-treated controls).

The final evidence for compound potential to work as an adjuvant needs to be provided by immunization studies. C57BL/6 mice (*n* = 5 mice per group) were subjected to immunization with 20 µg OVA/mice via IN route in the presence of 20% NACs or PBS vehicle, compared against negative control PBS IN instillations. The first immunization at the start of the studies was followed by repeat of the respective treatment at 4 weeks (booster immunization). In contrast with PBS treatment or OVA alone, NAC + OVA combination induced high titers of OVA-specific IgG antibodies in mouse sera, especially after second immunization (Figure 7A). NAC3 induced the highest anti-OVA IgG antibody titer after both first and second immunization, while NAC1, induced a still robust but nearly 50% lower antibody titer relative to NAC3 upon two immunizations. NACs triggered the induction of 2 types of IgG antibodies (IgG1 and IgG2c) in contrast to OVA-only vaccinated group that produced low albeit detectable amounts of IgG1 only (Figure 7B). Again, NAC3 seems to be most potent in the induction of both types IgG in the sera. We measured specific anti-OVA IgA in the serum which were shown to have an important role in dealing with pathogens like rotaviruses [43,44] and can be used as indirect measure for intestinal immunity [45]. Highest titers were measured for mice vaccinated with NAC2 + OVA and NAC3 + OVA, both groups had higher specific anti-OVA IgA titers than the group vaccinated with OVA only (Figure 7C). Finally, IgE anti-OVA antibody titers were below limiting of detection in sera in either of the treatment groups, suggesting that the NACs have not induced undesirable, IgE class switch following the immunization with OVA. Therefore, all 3 NACs showed true adjuvant properties in model antigen immunization, with NAC3 showing the strongest effect as an adjuvant.

## 4. Discussion

There is an urgent need for more convenient and less invasive vaccines such as intranasal vaccines. However, the development of such vaccines is limited by the need of appropriate adjuvants designed specifically for intranasal administration. Here, we developed a set of emulsion-based NACs and tested their biophysical and biological properties with the goal of selecting the best candidates for future development of new-generation adjuvants suitable for nasal vaccine administration.

The size of NAC droplets and their size distribution have great importance since they affect emulsion rheology, pharmacokinetics, stability and biological properties [33]. The droplet size for the tested NACs is at the range of 405.5 to 623.6 nm. Regarding ZP of NACs, they varied from 30.4 to 64.4 mV. Our data demonstrate that among NACs components the cationic detergent type determines ZP. It was previously reported that particles that were positively charged were better phagocytized [32], thus, NACs charge may facilitate the sampling of given antigen. All tested NACs enhanced antigen uptake by dendritic cells to the same degree (Figure 5A). Moreover, NACs enhanced antigen uptake by airway epithelium cells, the observation is important since inadequate antigen absorption by mucosal epithelium poses a severe barrier to nasal vaccine delivery [46]. We showed that NAC1 and NAC3 increased DQ-OVA uptake by TC-1 cells when incubated with the protein (Figure 5B). Interestingly, DQ-OVA uptake was proportional to the size of nanodroplets, NAC3 with the biggest droplets enhanced the uptake the most. Also, NAC3 droplets possess the highest ZP (63.5 mV). Our observations are in agreement with previously published data indicating that nanoparticles with higher ZPs (50 mV) interacted better with eukaryotic cells than particles with lower ZPs (20 mV, −20 mV and −30 mV) [47].

Facilitated antigen uptake by NACs can increase capacity of the antigen presenting to immune cells. It is important in the context of increasing the bioavailability of polar drugs, that is very weak due to the low epithelium permeability [46]. The drugs can cross the membrane by one of the following routes; by antigen sampling by dendritic cells mentioned above, through the cell interior or tight junctions [48]. Here, we presented the promoting effect of NACs on DQ-OVA engulfment by dendritic and airway epithelial cells. The mechanism of this observation is not fully understood. Based on the study of other authors [49], we lean toward the possibility that the interaction between the cell membrane and nanoadjuvant candidate can change the cell membrane fluidity and thereby promotes efficient uptake. In the future, the in vivo studies will dissect the unique immunomodulatory NACs properties. Furthermore, the ZP is an important factor for the cellular uptake. Moderately positive charge of NAC (between 30.6 mV and 63.5 mV) is one of the desired features of new mucosal adjuvants. The cationic emulsions have been previously shown to be stable in the presence of physiological cations, interacted in vivo with negatively charged biological membranes leading to an enhanced permeability, drug uptake and site-specific targeting [16,32]. Mucosal vaccines should effectively expose antigens to immunocompetent cells leading to promote immune response against the antigen.

Another way to facilitate antigen uptake by mucosa is to extend its residence time in nasal cavity. Positively charged NACs bind to mucin which was shown to be beneficial for prolonged contact of mucus with an antigen, thus, facilitating antigen sampling by epithelial cells [37]. Mucin is a negatively charged protein, strongly cross-linked, which binds moisture and acts as a lubricant [50]. It was previously shown that mucoadhesion can be achieved by using charged polymers as a carrier for vaccine antigens [51]. All tested NACs interacted electrostatically with mucin and the interaction was manifested by the decreasing ZPs, from positive to negative (Figure 1). However, the size each of tested NACs did not change upon mucin binding. It should be noted that mucin is a heterogenic protein with sizes between 10 and 300 nm and molecular weights from 200 kDa to 200 MDa [52], which makes it difficult to draw conclusions about the character of its interaction with NACs based on size measurement results only. However, given the hydrophilic nature of this protein, we are unlikely to expect mucin to penetrate into the nanoadjuvant droplets, which are lipophilic. Hence, there is no increase in NACs droplets size. It is different in the case of NAC interactions with ovalbumin, which has a defined size (molecular weight is 45 kDa) and is less hydrophilic than mucin. The addition of OVA to NACs causes a significant enlargement of the droplets (Figure 6), which indicates that the protein is loading inside the droplets thus affecting their size. This results of the interaction between NAC and mucin or OVA seems very surprising and begs for further detailed studies using transmission electron microscopy (TEM) [31]. However, in this study, OVA was used as a model antigen and this kind of adjuvant-antigen interaction should be investigated in the case of each new vaccine antigen of interest.

NACs charge is a feature of their biological effect on the cells. Since positively charged droplets might be toxic, we tested the effect of NACs on viability of the cells (Figure 2). We were able to determine concentrations of NACs that were safe for macrophages, dendritic cells and epithelial cells, as well as sub-cytotoxic concentrations that later were shown to have interesting immunostimulatory effects. We showed that the mouse airway epithelial cell line (TC-1) appeared to be more resistant to the NACs cytotoxic effect during shorter incubation time than macrophages or dendritic cells. It is a very interesting observation which may indicate that epithelial cells may be more resistant to exogenous substances than macrophages, as they are in the first line of contact. This observation needs to be taken into consideration when planning next experiments. One of the limitations of this study is the lack of positive control, for example doxorubicin, that would show the effect of total cell damage [53].

Sub-cytotoxic effect of NACs on the epithelial cells may induce the danger signal and evoke the immune response. It has been previously reported that dying cells stimulate dendritic cells to mature and present foreign antigens to other immune cells like T lymphocytes [54,55]. NACs do not contain any immunostimulators themselves, therefore they do not induce any proinflammatory cytokines. Albeit, when NACs are administrated to cells with sub-activating concentrations of LPS they can induce up to 3 times more TNF-α comparing to cells incubated with LPS alone (Figure 3). We did not detect changes in the concentrations of other cytokines. It has been previously documented that emulsion-based adjuvants, in dendritic cells, can activate TLR2 and 4 [56], which are the receptors for LPS. In this study, we incubated cells for 24 h (the same time as Bielinska et al.) and observed potentiated TNF-α response in macrophages. Therefore, activation of TLR2 and 4 might be a potential mechanism by which BMDM treatment with NACs makes cells more sensitive to increase their response to LPS by the production of TNF-α. Another possible mechanism of action of NACs might be connected with their influence on these receptors’ expression. We have shown that the addition of NACs increases TLR4 expression after LPS stimulation (Figure 4). TLR4 agonists are already extensively researched as novel adjuvants such as the approved Monophosphorylated Lipid A and aminoalkyl glucosaminide phosphates [57]. However, little is known about compounds affecting increased expression of this receptor. Glucopyranosyl Lipid A formulated with Stable Emulsion (GLA-SE) was shown to increase expression of genes implicated in TLR4 signaling resulting in stronger immune response than alum [58]. It would seem that an increase in receptor expression would lead to an increase in its presence on the cell surface and an enhancement of the response to a given formulation. Our observations show that NACs may have a dual effect, firstly, through controlled cytotoxicity they induce the formation of endogenous danger signals. Secondly, they amplify the response to these signals by increased absorption and induction of expression of TLRs. In the future, more detailed studies are warranted to address this topic, but the resulting adjuvant activity by NACs has been confirmed by us in in vivo experiment.

We vaccinated intranasally C57BL/6 mice with 20% NACs and 20 µg/mouse OVA. This experiment showed that vaccines consisting of NACs and OVA administered IN are safe. Mice did not show any signs of distress after immunization. A detailed study of the NACs influence on nasal mucosa in vivo is underway. NACs administrated IN with OVA induced high titers of OVA-specific IgG antibodies after just two vaccinations. Especially NAC3, having the highest ZP and size, induces considerable amounts of specific IgG and high levels of IgG1 and some IgG2c antibodies as well (Figure 7). It was previously shown that the response of IgG isotypes (IgG1 and IgG2a/IgG2c) is essential for vaccine efficiency in case of some viruses like influenza [59]. This shows that NACs have the potential to be effective intranasal adjuvants. We measured the levels of specific anti-OVA IgA in the serum that can be a prognostic marker for IgA response in the mucosal sites and showed that NAC2 and 3 induce higher specific IgA titers than OVA alone. There were no OVA-specific IgE antibodies in the serum of vaccinated mice which indicates that OVA administrated in NACs did not induce an allergic response. Another aspect is to investigate how NAC influences the induction of a cellular response, e.g., the use of SIINFEKL (OVA) peptide in combination with NAC. The SIINFEKL peptide is presented by dendritic cells via MHC-I receptors and recognized by surface receptors present on T lymphocytes. The degree of loading of the MHC-I receptors with the peptide can be easily monitored by cytofluorimetric measurements [60]. Such research will be carried out by us in the future. One of the limitations of the study is lack of data for each mouse, a pooled sera was used instead. However, this preliminary experiment shows the great potential of NACs and their adjuvant properties are the focus of our further research. To sum up, NACs elicits versatile humoral response against given antigen without inducing allergy associated IgE response.

## 5. Conclusions

The study results support the notion that NAC1, 2 and 3 have potential to be suited as components of the mucosal vaccines. Our study demonstrated that NACs interact with OVA protein, exhibit bio-adhesion to mucin, potentiate cytokine production, TLR4 expression and enhance protein sampling by dendritic and epithelial cells. We showed their effectiveness as adjuvants in in vivo studies, which promote us to design and manufacture the versatile nasal drug delivery carrier and adjuvant. The studies regarding mucosal response after vaccination with NACs is ongoing.

## Figures and Tables

**Table 1:** Percentage composition (*w*/*v*) of examined NACs.

Name	Oil (65%)	Organic Solvent (8%)	Nonionic Surfactant (5%)	Cationic Surfactant (1%)	Solvent Used for Surfactant Preparation (21%)
NAC1	PDMS	acetone	tyloxapol	BDMDDAC	water
NAC2	PDMS	ethanol	tyloxapol	BDMDDAC	water
NAC3	PDMS	ethanol	tyloxapol	CBP	water

Abbreviations used: BDMDDAC-benzyldimethyldodecyl ammonium chloride; CBP-cetylpirydinium bromide; PDMS–polydimethylsiloxane.

**Table 2:** Long-term stability of NACs. NACs were measured at time 0, 1, 2, 3, 4, 5, 6 and 12 months. (**A**) shows changes in size (nm) during 12 months of storage. PDI values are in the brackets. (**B**) shows changes in ZP (mV) during 12 months of storage. Average from five measurements ± SD.

**(A)**								
**Name**	**Size Measurements**							
**Months**	**0**	**1**	**2**	**3**	**4**	**5**	**6**	**12**
NAC1	534 ± 9.9 (0.31)	472.6 ± 6.5 (0.311)	512.1 ± 7.8 (0.362)	467.5 ± 4.8 (0.312)	472.2 ± 6.7 (0.274)	492.8 ± 8.1 (0.312)	506.6 ± 10.5 (0.354)	458 ± 4.9 (0.257)
NAC2	405.5 ± 9.2 (0.265)	386.2 ± 5.3 (0.254)	390.6 ± 6.1 (0.26)	370.9 ± 2.8 (0.211)	374.2 ± 3.5 (0.23)	367.9 ± 2.9 (0.209)	394.3 ± 4.8 (0.246)	369.5 ± 2.4 (0.217)
NAC3	623.6 ± 6.0 (0.468)	565 ± 18.3 (0.387)	542.8 ± 5.9 (0.349)	580.4 ± 3.3 (0.4)	553.9 ± 3.3 (0.359)	604.4 ± 17.9 (0.4)	565.7 ± 8.7 (0.367)	551.8 ± 12.3 (0.334)
**(B)**								
**Name**	**Zeta Potential Measurements**							
**Months**	**0**	**1**	**2**	**3**	**4**	**5**	**6**	**12**
NAC1	30.4 ± 0.4	39.1 ± 0.4	36.5 ± 1.1	39.9 ± 0.6	35.8 ± 0.5	40.4 ± 0.6	35.1 ± 0.2	43.6 ± 0.5
NAC2	31.6 ± 0.7	38.5 ± 0.3	35.7 ± 0.4	33.6 ± 0.3	32.3 ± 0.5	38.1 ± 0.7	33.6 ± 0.2	39.5 ± 0.3
NAC3	64.4 ± 1.0	66.7 ± 1.1	64.3 ± 0.2	70.8 ± 0.6	65 ± 0.5	65.8 ± 0.9	62.4 ± 0.6	56.4 ± 0.2

## Data Availability

The data is available from the corresponding author upon request.
